# Dysbiosis of Oral Microbiota During Oral Squamous Cell Carcinoma Development

**DOI:** 10.3389/fonc.2021.614448

**Published:** 2021-02-23

**Authors:** Purandar Sarkar, Samaresh Malik, Sayantan Laha, Shantanab Das, Soumya Bunk, Jay Gopal Ray, Raghunath Chatterjee, Abhik Saha

**Affiliations:** ^1^School of Biotechnology, Presidency University, Kolkata, India; ^2^Human Genetics Unit, Indian Statistical Institute, Kolkata, India; ^3^Department of Life Sciences, Presidency University, Kolkata, India; ^4^Department of Oral Pathology, Dr. R Ahmed Dental College and Hospital, Kolkata, India

**Keywords:** oral squamous cell carcinoma, 16S rRNA sequence analysis, oral microbiology ecology, dysbiosis, human papillomavirus-16

## Abstract

Infection with specific pathogens and alterations in tissue commensal microbial composition are intricately associated with the development of many human cancers. Likewise, dysbiosis of oral microbiome was also shown to play critical role in the initiation as well as progression of oral cancer. However, there are no reports portraying changes in oral microbial community in the patients of Indian subcontinent, which has the highest incidence of oral cancer per year, globally. To establish the association of bacterial dysbiosis and oral squamous cell carcinoma (OSCC) among the Indian population, malignant lesions and anatomically matched adjacent normal tissues were obtained from fifty well-differentiated OSCC patients and analyzed using 16S rRNA V3-V4 amplicon based sequencing on the MiSeq platform. Interestingly, in contrast to the previous studies, a significantly lower bacterial diversity was observed in the malignant samples as compared to the normal counterpart. Overall our study identified *Prevotella, Corynebacterium*, *Pseudomonas*, *Deinococcus* and *Noviherbaspirillum* as significantly enriched genera, whereas genera including *Actinomyces*, *Sutterella*, *Stenotrophomonas*, *Anoxybacillus*, and *Serratia* were notably decreased in the OSCC lesions. Moreover, we demonstrated HPV-16 but not HPV-18 was significantly associated with the OSCC development. In future, with additional validation, this panel could directly be applied into clinical diagnostic and prognostic workflows for OSCC in Indian scenario.

## Introduction

Oral squamous cell carcinoma (OSCC), a subset of head and neck squamous cell carcinoma (HNSCC), is the most common oral malignancy, representing approximately 90% of all cancers in the oral cavity (1, 2). It is the sixth most common cancer worldwide and every year around 400,000 new cases are diagnosed (3, 4). The number of newly diagnosed cases is predicted to increase by 62% in 2035 (5). The prevalence of oral cancer is highest in India and it represents most prevailing cancer in male population and fifth most common cancer among women (6, 7). Despite easy accessibility of the oral cavity during physical examination as well as several technological advancements in surgical procedures in addition to adjuvant radiotherapy and chemotherapy, due to the lack of early diagnosis based on appropriate molecular markers, OSCC patients are often diagnosed at more advanced stages, leading to poor survival outcomes. The overall 5-year survival rate of OSCC patients is roughly 50% across the globe (8–10). Thus, early detection, identification of biomarkers and understanding the role of various etiological agents can significantly improve the current situation of OSCC treatment. In developing countries like India, excessive tobacco usage including smoking, chewing betel quid and areca nut along with alcohol consumption are the major risk factors for OSCC development (7, 11). However, oral cancer often arises in patients without a history of tobacco usage or alcohol consumption, indicating contribution from other potential risk factors including genetic/epigenetic alterations or microbial infection (2, 6, 12). A number of oncogenic viruses including high risk human papillomavirus (HPV) genotypes and Epstein-Barr virus (EBV) have been identified as infectious etiological agents for OSCC (13–15). However, association of these oncoviruses with OSCC development is not strong, contributing approximately 20% of all oral cancers (13, 14). Thus, identification of other microbial factors influencing OSCC development is warranted.

Human body harbors a plethora of microbial species referred to as ‘commensal microbiota’ including bacteria, yeast, fungi, protozoa, archaea and viruses and develops a symbiotic ecosystem without eliciting a decimating immune response. However, alterations of the microbiome architecture (dysbiosis) often lead to a variety of human diseases including cancer (16, 17). The advent of next-generation sequencing technologies for example the 16S rRNA gene amplicon based sequencing has allowed an affordable and culture-free approach of identification of overall bacterial composition in cancerous lesions and its effect on the progression of the disease (18). As one of the prime territories of microbiome in human body, the oral cavity contains distinct niches with dynamic microbial communities (19). Oral microbial ecology is a critical factor in controlling both human physiology and pathophysiology. The oral microbiome and their produced metabolites translocate through gastrointestinal tract or due to periodontal pocket ulceration can affect various distant tissues and are associated with the development of a number of diseases like cardiovascular disorder, diabetes, rheumatoid arthritis and premature birth (20–23). The dysbiosis of oral microbiome is associated with a number of clinical symptoms that ranges from dental caries, periodontal disease to oral cancer (24–28). Importantly, chronic periodontitis has also been suggested as potential risk factor for the onset of oral pre-cancerous and cancerous lesions (27). There is, however, no consensus among reports regarding microbiome signature associated with the development of OSCC. For example, Schmidt et al. demonstrated depletion of Firmicutes and Actinobacteria in a study of 15 oral cancer patients (29), while Mager *et al*. using DNA hybridization technique reported elevation of *Capnocytophaga gingivalis*, *Provatella melaningtoenica*, and *Streptococcus mitis* in the saliva of OSCC patients (30). Recently, Zhao *et al*. demonstrated that a cluster of periodontitis associated taxa such as *Fusobacterium, Dialister, Peptostreptococcus, Filifactor, Peptococcus, Catonella*, and *Parvimonas* was enriched in OSCC lesions (31). AI-hebshi et al. reported that several inflammatory bacterial species including *Pseudomonas aeruginosa* and *Fusobacterium nucleatum* are elevated in OSCC patients’ samples (32). Another report by Lee *et al*. demonstrated that salivary microbiome particularly five genera including *Bacillus*, *Enterococcus*, *Parvimonas*, *Peptostreptococcus*, and *Slackia* significantly varied between samples from the epithelial precursor and OSCC lesions (33), indicating a potential prognostic marker for OSCC development. Börnigen et al. identified a number of differentially abundant genera in oral cancer samples specifically *Dialister* as enriched and *Scardovia* as depleted (34). Overall, given the diversity of identified microbiome composition as well as limited number of samples, more in depth investigations with larger-scale epidemiologically designed cohorts are warranted to assess the role of microbiome dysbiosis in OSCC development.

Despite the highest oral cancer incidence in India, till date no efforts have been made in understanding the oral microbial imbalance during OSCC development among Indian patients. Here, in an aim to explore OSCC-associated bacterial composition fifty OSCC lesions and their anatomically matched normal tissue regions was profiled using 16S rRNA gene amplicon based sequencing by targeting the hypervariable V3-V4 region. Our analyses revealed while top five genera such as *Prevotella, Corynebacterium, Pseudomonas, Deinococcus* and *Noviherbaspirillum* were significantly enriched, while genera including *Actinomyces, Sutterella, Stenotrophomonas, Anoxybacillus, and Serratia* were notably depleted in the OSCC lesions as compared to matched control adjacent tissue samples. In sum, our results provided evidence of alterations of oral bacterial community during OSCC development and indicated the possibility of utilizing the identified microbiome signature as prognostic marker of oral malignancies in patients of Indian subcontinent.

## Materials and Methods

### Ethics Statement

The study was approved by the Institutional Ethics Committee for Human Research, Indian Statistical Institute, Kolkata, India. Written informed consent was obtained from all participants and all methods in this study were performed in accordance with the relevant guidelines and regulations.

### Sample Information

After the clinical diagnosis of oral squamous cell carcinoma (OSCC) the patients from Dr. R Ahmed Dental College and Hospital, were enlisted for the study. 50 patients were included in the study after confirmation of well-differentiated squamous cell carcinoma from histopathological reports. All participants were not on any local or systemic antibiotics prior to sample collection. Tissue samples were collected by incisional and 3 mm punch biopsy sample collection method from both the regions of cancerous lesions (N=50) and the adjoining clinically uninvolved normal area (matched control, N=50) for each of the 50 patients recruited in this study. A portion of the tissue samples were collected in RNA Later (Invitrogen, Thermo Fisher Scientific Inc., Waltham, MA, USA) and stored at -80°C for future use. Another portion was fixed in the formalin and used for histopathological evaluations.

### DNA Extraction

DNA was isolated from the cancerous lesion and adjacent unaffected normal tissue regions using DNeasy Blood and Tissue Kit (Qiagen, Hilden, Germany) according to manufacturer’s protocol. The quality and quantity of isolated DNA was determined by the A260/280 ratio using Synergy H1 Multimode Microplate Reader (BioTek Instruments, Inc., VT, USA). DNA samples were frozen at -20°C for further analysis. Approximately 50 ng of genomic DNA from each sample was used for 16S rRNA V3-V4 amplicon sequencing.

### 16S Ribosomal RNA Sequencing and OTU Assignment

16S ribosomal RNA (rRNA) amplicon sequencing for metagenomics studies was performed on a MiSeq platform (Illumina, San Diego, CA, USA) using 2×250 bp chemistry. Clonal libraries for 16S rRNA V3-V4 hypervariable region were prepared using NEBNext Ultra DNA Library preparation kit (New England Biolabs, Ipswich, MA, USA) with the forward primer (5′-CTTTCCCTACACGACGCT CTTCCGATCTACGGRAGGCAGCAG-3′) and the reverse primer (5′-GGAGTTCAGACGTGTGCT CTTCCGATCTTACCAGGGTATCTAATCCT-3′). The amplicons were subjected to a number of enzymatic reactions for end-repairing, dA-tailing followed by adapter ligation and cleaning up using Solid Phase Reversible Immobilization (SPRI) technology (Beckman Coulter, Indianapolis, IN, USA). The adapter ligated fragments were indexed by limited PCR cycle to generate final libraries for paired-end sequencing. The concentration of the purified amplicons was measured using Qubit fluorometer (Thermo Fisher Scientific Inc., Waltham, MA, USA) and the quality was checked using Agilent 2100 Bioanalyzer (Agilent Technologies, Santa Clara, CA, USA). The multiplex amplified libraries were pooled in equimolar concentrations with unique indices, mixed with 15% PhiX control and sequenced using MiSeq reagent kit v2 (Illumina, San Diego, CA, USA) according to manufacturer’s instruction.

The raw FASTQ sequencing files were further processed after checking base quality, base composition and GC content using FASTQC toolkit. The targeted amplicons were filtered out from other superfluous sequences by detecting the specific conserved region. Forward and reverse reads were stitched together with a minimum overlap of 30 bp and maximum overlap of 250 bp. De-replication was performed using USEARCH (35) (v11) for the identification of unique sequences and chimera sequences were filtered out using the UCHIME (36) algorithm in USEARCH package. Sequences that had a similarity of 97% were grouped together under a single operational taxonomic unit (OTU) against the GreenGenes database (release 2013-08: gg_13_8_otus) using UPARSE (37) method. The taxonomy classification and relative abundance assignments were performed using ‘Quantitative Insights Into Microbial Ecology’ (QIIME v. 1.9.0) (38) pipeline and singletons were discarded from the dataset to minimize the effect of low abundance sequences. To confirm the annotation, the resulting OTU representative sequences were then searched against the Ribosomal Database Project naïve Bayesian classifier (RDP 10 database, version 6) (39, 40) database, using the online program SEQMATCH (40). The taxonomy classifications at the phyla, order, family, genera and species level were performed using the GreenGenes and RDP databases.

### Diversity and Bacterial Enrichment Analyses

MicrobiomeAnalyst (41) was used for statistical analysis. The α-diversity indexes including observed OTU numbers, Chao index, Simpson index, and Shannon index and the β-diversity – Bray-Curtis dissimilarity measurements were calculated. Evolutionary relation of the genera unique to the OSCC samples and the normal counterparts were analyzed and a cladogram was generated using Galaxy (42). The variation in genera as well as the unique bacterial composition in the normal and OSCC samples was identified using Random Forest (43) classification analysis within MicrobiomeAnalyst (41).

To estimate β-diversity, un-weighted and weighted UniFrac distances by Bray-Curtis method were calculated from the OTU abundance and utilized in Principal Component Analysis (PCoA) to analyze the unique clustering genera for the normal and OSCC affected tissue samples. PERMANOVA (44) algorithm on un-weighted and weighted UniFrac distance matrices was applied to generate PCoA plots. The differential abundances of OTUs and specific OTU enrichment between OSCC samples and matched controls were determined using LEfSe based on Kruskal–Wallis H test. Pairwise OTU enrichment analysis was performed to specifically identify the OTU abundance in each sample pair by comparing their true abundance values in the OSCC sample and its normal counterpart.

### Functional Prediction of Distinct Bacterial Communities

Functional compositions of the bacterial communities among two different groups were predicted using Phylogenetic Investigation of Communities by Reconstruction of Unobserved States (PICRUSt) (45) according to the Kyoto Encyclopedia of Genes and Genomes (KEGG) database (46).

### Real-Time PCR Primer Designing and Data Output

Primer-BLAST tool (https://www.ncbi.nlm.nih.gov/tools/primer-blast/) in National Center for Biotechnology Information (NCBI) database was used to design primers for real-time quantitative PCR (qPCR) analyses. Primers for conserved sequence of bacterial 16S rRNA gene, human Glyceraldehyde 3-phosphate dehydrogenase (GAPDH) gene and human oncogenic viruses HPV16, HPV18 and EBV are listed in **Table S2**. qPCR primers were obtained from Integrated DNA Technologies, Inc. (Coralville, IA, USA). The optimum primer melting temperature (Tm) was chosen at 60°C and the maximum GC content was kept at 55%. qPCR analysis was performed using iTaq Universal SYBR Green Supermix (BIO-RAD, Hercules, CA, USA) in CFX Connect Real-Time PCR detection System (BIO-RAD, Hercules, CA, USA) with the following thermal profile – one cycle: 95°C for 10 min; 40 cycles: 95°C for 10 s followed by 60°C for 10 s; and finally the dissociation curve at – 95°C for 1 min, 55°C 10 s, and 95°C for 10 s. Unless and otherwise stated, each sample was performed in duplicate and calculation was made using a −ΔCT method to quantify relative abundance compared with human genomic GAPDH control. The -ΔC_t_ values of each OSCC samples and their match controls were plotted using GraphPad Prism 8.0.1.

## Results

### Subject Characteristics and Oral Microbiota Profiling by 16S rRNA V3-V4 Amplicon Sequencing

To investigate changes in the oral microbiome associated with OSCC development, we prospectively collected cancerous lesions and anatomically matched adjacent normal tissue samples from four OSCC patients. Prior to proceeding into 16S rRNA amplicon based metagenomics studies, a preliminary verification was conducted for confirmatory presence of bacterial species in the isolated genomic DNA from the clinical samples. A real time quantitative PCR (qPCR) assay was performed using primer against the 16S rDNA conserved region. Excellent PCR amplification curves and -ΔC_t_ values calculated against the housekeeping human GAPDH gene demonstrated the presence of bacterial species in both OSCC samples and their normal counterparts (**Figure S1**). To expand and substantiate our initial observations, we further collected another 46 pair of OSCC lesions and adjacent healthy tissue samples. The bacterial DNA was isolated from all 50 pair specimens (**Table S1**), followed by PCR amplification targeting the 16S rRNA V3–V4 hypervariable region. The 16S amplicons were purified, and a second round of index PCR was performed. The multiplex amplified libraries were pooled at equimolar concentrations and sequenced on an Illumina MiSeq platform. A total of 477,411 raw sequences were generated and after quality trimming and chimera checking, 322,656 high quality sequences were recovered for downstream analysis, with an average of 3,226 reads, ranging from 1,703 to 11,411 reads per sample.

### Genera Abundance and Diversity of Oral Microecology Were Depleted During OSCC Development

**Figure 1:**
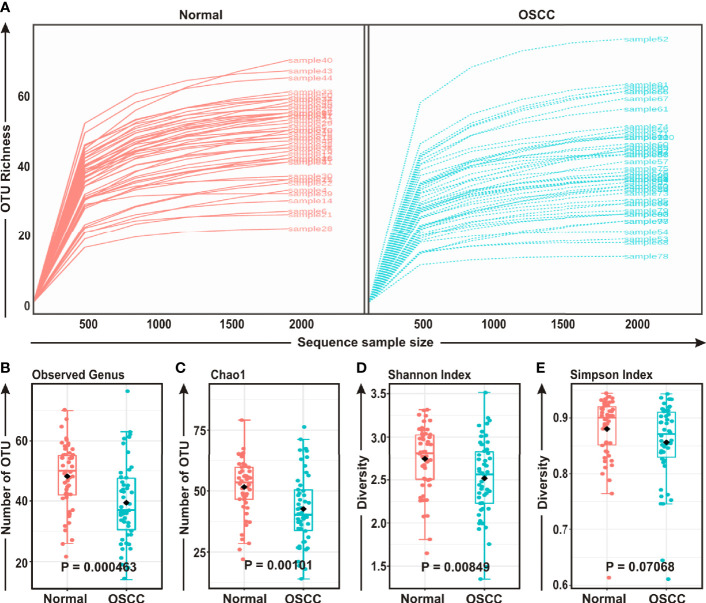
Comparison of oral microbiome compositions in the oral squamous cell carcinoma (OSCC) lesions and contralateral normal healthy groups. **(A)** Rarefaction analysis of bacterial 16S rRNA gene sequences of normal (red) and OSCC lesions (blue). Each line represents one sample. **(B–E)** Box-Whisker plots of **(B)** Observed operational taxonomic units (OTUs), **(C)** Chao 1 and **(D)** Shannon Index, **(E)** Simpson Index respectively.

FASTQ files generated in 16S rRNA gene sequencing of all 50 samples containing high quality sequences were further analyzed using MicrobiomeAnalyst, a web-based online tool for comprehensive statistical analysis of microbiome data (41). Rarefaction plot generated from the mapped reads indicated a clear difference of the OTU (Operational Taxonomic Unit) richness at the genus level between OSCC and normal samples (**Figure 1A**). Most of the samples, though not entirely, reached a saturated plateau phase, indicating further sequencing would possibly generate additional genera in those samples (**Figure 1A**). The average data of rarefaction plot demonstrated elevated genus richness in anatomically matched controls (samples 1–50) as compared to the paired contralateral OSCC lesions (samples 51–100), signifying a potential loss of certain genera during OSCC progression (**Figure 1A****)**. The OTU richness was further analyzed by calculating alpha diversity of oral microbiota, indicating the differences and similarities of the identified genera between the two sample groups (**Figures 1B–E**). The Observed genus (*p* = 0.000463) and Chao1 index (*p* = 0.00101) indicated that the OTU richness was significantly depleted in the OSCC samples as compared to the matched controls. The diversity estimator Shannon index (*p* = 0.00849) indicated that relative diversity of bacterial genera was significantly decreased in cancerous lesions in contrast to the normal tissue sections (**Figures 1B–D respectively**). A similar trend of depletion in relative diversity of bacterial genera in OSCC samples as compared to the normal counterparts was also observed by employing Simpson index, although the data was not statistically significant (*p* = 0.070680) (**Figure 1E**).

### Anatomically Matched OSCC and Normal Samples Comprised of Distinct Microbiome Composition

**Figure 2:**
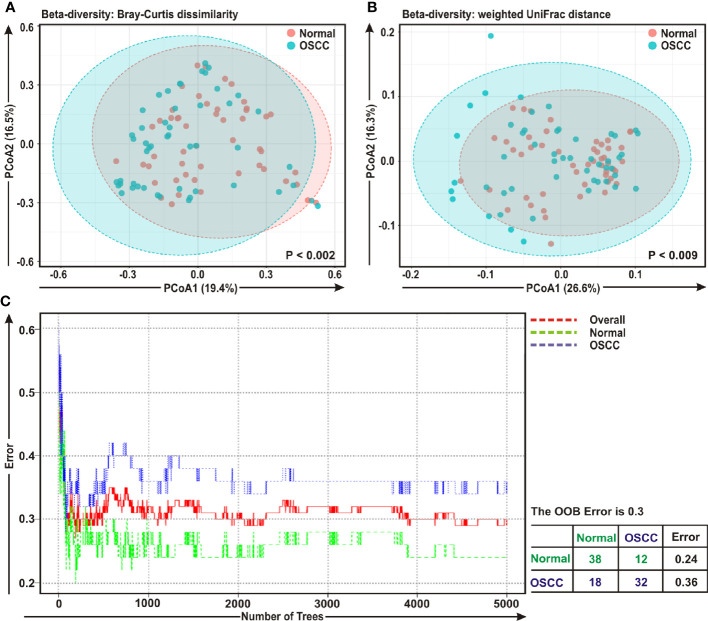
Beta diversity analyses among cancerous and normal samples. Principal Component Analysis (PCoA) plots based on **(A)** Bray-Curtis dissimilarity and **(B)** weighted UniFrac distance matrices with respect to the bacterial abundance and composition among oral squamous cell carcinoma (OSCC) lesions and adjacent normal tissue samples. **(A)** Axis 1 (PCoA1): 19.4% of variation explained. Axis 2 (PCoA2): 16.5% of variation explained. **(B)** Axis 1 (PCoA1): 26.6% of variation explained. Axis 2 (PCoA2): 16.3% of variation explained. **(C)** The error plots identified from random forest classification analyses. Red-line indicates the overall genera present in both OSCC and normal samples, green-line indicates the distinct genera present in the normal samples and the blue-line indicates the specific genera present in the OSCC lesions.

The beta diversity indicates the difference in the composition of bacterial community among different sample groups (47). To estimate β-diversity, weighted UniFrac distances as well as Bray-Curtis dissimilarity metric were calculated from the OTU abundance and utilized in Principal Component Analysis (PCoA) (47). Permutational Multivariate Analysis of Variance (PERMANOVA) (44) algorithm on Bray-Curtis dissimilarity and weighted UniFrac distance matrices was applied to generate PCoA plots (**Figures 2A, B**, respectively). The bacterial communities in the cancerous lesions and the anatomically matched controls clustered discretely, suggesting the overall structures of the bacterial communities in the groups were significantly different (*p* < 0.002 and p < 0.009, respectively in two analyses) (**Figures 2A, B****)**.

A ‘Random Forest’ algorithm (43) was applied to further confirm the difference in bacterial community among the OSCC samples and anatomically matched healthy controls (**Figure 2C**). The decision trees extracted from the random forest classification identified distinct bacterial composition in diseased samples when compared with the normal counterparts. In the error plots identified from random forest analyses, while the red-line indicated the overall genera present in both OSCC and normal samples, green-line indicated the distinct genera present in the normal samples and the blue-line indicated the specific genera present in the OSCC lesions (**Figure 2C****)**. Moreover, in the total of 50 OSCC samples, 32 samples revealed unique and 18 samples demonstrated overlapping genera; whereas in case of contralateral paired 50 normal tissue samples, 38 samples exhibited unique and 12 samples showed overlapping genera (**Figure 2C****)**.

### Phylogenetic Analysis Revealed Variations Among Common and Distinct Taxa in OSCC Lesions and Anatomically Matched Healthy Controls

**Figure 3:**
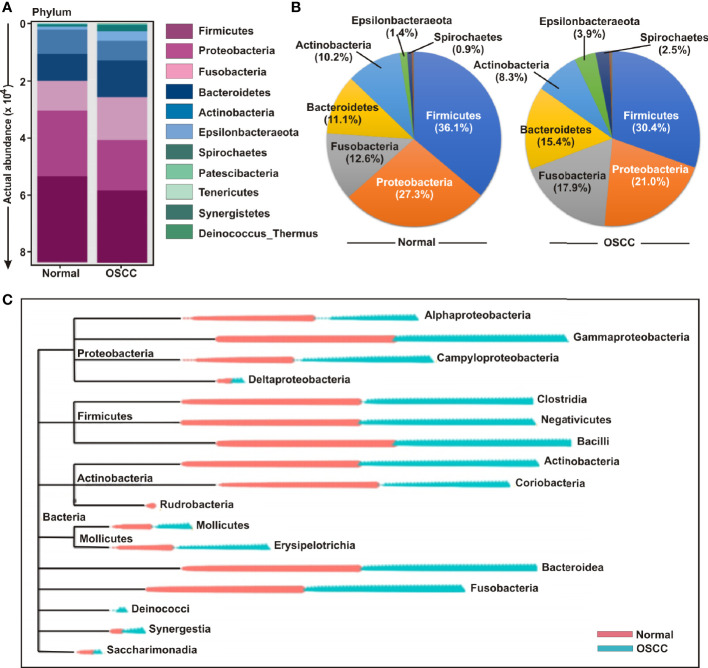
Composition of bacterial communities across samples at the phylum and genus levels. **(A)** Actual and **(B)** relative abundance of bacterial communities at phylum level of OSCC lesions and anatomically matched controls. **(C)** Phylogenetic tree with operational taxonomic unit (OTU) abundances between OSCC and normal samples.

The bacterial communities in the OSCC lesions and the anatomically matched healthy controls were first analyzed at phylum level (**Figures 3A, B****)**. The top five most abundant phyla including *Firmicutes, Proteobacteria, Fusobacteria, Bacteroidetes*, and *Actinobacteria* collectively comprised of 97.3% and 93% of all sequences in matched controls and OSCC lesions, respectively (**Figure 3B****)**. *Firmicutes* was the most abundant phylum in all samples, accounting for 36.1% of sequences in matched controls and 30.5% in OSCC lesions. In contrast, the abundances of the other detected phyla, including *Epsilonbacteraeota*, *Spirochaetes*, *Patescibacteria*, *Tenericutes*, *Synergistetes*, and *Deinococcus*, were less than 4.0%, ranges from 0.3% to 3.96%. While abundance of phyla including *Firmicutes, Proteobacteria* and *Actinobacteria* were reduced, *Fusobacteria, Bacteroidetes*, *Epsilonbacteraeota*, and *Spirochaetes* were elevated in OSCC lesions compared to normal healthy controls (**Figures 3A, B****)**. At the genus level, *Streptococcus*, *Leptotrichia*, *Fusobacterium*, *Serratia*, *Neisseria*, *Haemophilus*, *Gemella*, *Campylobacter*, *Veillonella*, *Capnocytophaga*, *Prevotella*, *Porphyromonas*, *Rothia*, *Bifidobacterium*, and *Bacteroides* were the fifteen most abundant genera, comprising of 13.63%, 8.77%, 6.42%, 6.14%, 4.97%, 4.28%, 2.96%, 2.67%, 2.47%, 2.24%, 2.12%, 1.87%, 1.87%, 1.85%, and 1.84% sequence coverage, respectively (**Figures S2A-C**). Of all genera detected, 137 taxa were found common in all samples, while 26 and 29 taxa were distinctly present OSCC lesions and anatomically matched controls, respectively (**Figure S2D****)**. The shared genera among OSCC lesions and normal samples collectively represented more than 97.0% of all detected sequences in oral microbiota (**Figure S2****)**.

In order to further determine the differential presence and abundance of oral microbial community a phylogenetic tree was generated up to class level by MicrobiomeAnalyst (**Figure 3C**). The result demonstrated that Rudrobacteria class under phylum Actinobacteria was exclusively present in normal samples, whereas Deinococcus phylum was exclusively present in OSCC lesions (**Figure 3C**). A number of bacterial genera under the classes of Gammaproteobacteria, Clostridia, Negativicutes, Bacilli, Actinobacteria, Bacteroidea and Fusobacteria demonstrated an elevated abundance in both groups without significant deference in distribution between OSCC lesions and anatomically matched normal samples (**Figure 3C**).

### Enrichment Analysis Identified Unique Genera for OSCC and Adjacent Normal Tissue Samples

**Figure 4:**
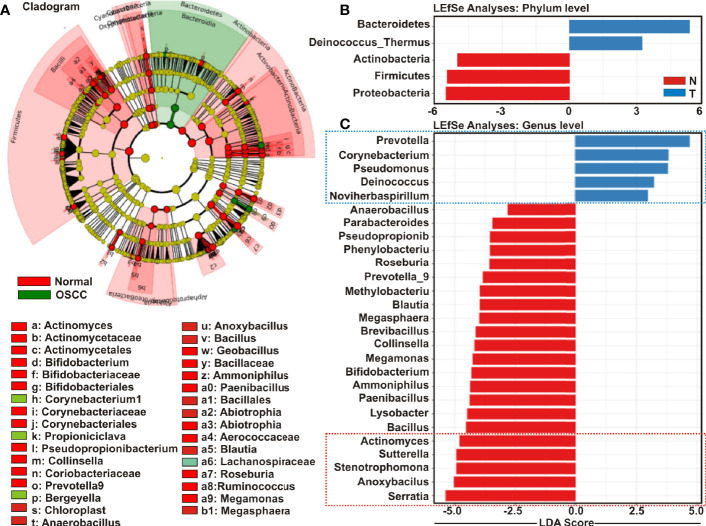
Cladogram and enrichment analysis among among oral squamous cell carcinoma (OSCC) lesions and clinically normal tissue samples. **(A)** Cladogram for phylogenetic relation of Normal and OSCC genus. Cladogram was constructed using the Linear Discrimination Analysis (LDA) Effect Size (LEfSe) method to indicate the phylogenetic distribution of bacteria that were significantly enriched in the tumor and normal groups. LDA scores showed significant bacterial differences within groups OSCC and clinically normal counterparts at the **(B)** phylum level and **(C)** genus level.

Cladistic analysis allows for a precise definition of biological classification in which organisms are categorized in ‘clades’ (or groups) based on the most recent common ancestor and are best depicted by cladogram models indicating the relation between the different levels of clades in multiple sample groups. Identification of differential microbial ecology at phylum level would thus further facilitate to correlate their potential effect on OSCC progression. A cladogram was generated using Galaxy (42), a web-based platform for bioinformatic analysis, to visualize significantly enriched bacteria taxa identified in OSCC lesions and anatomically matched adjacent control tissue samples (**Figure 4A****)**. The result demonstrated that while *Bacteroidetes* phylum was phylogenetically predominant, a number of phyla including *Firmicutes* and *Actinobacteria* were depleted in the cancerous lesions as compared to the healthy controls (**Figure 4A**).

Linear Discrimination Analysis (LDA) Effect Size (LEfSe) algorithm allows identifying high dimensional biomarkers among multiple study groups (48). To identify the distinguishing taxa within OSCC lesions and matched controls, we applied LEfSe method (**Figures 4B, C****)**. At the phylum level, *Bacteroidetes* and *Deinococcus* were significantly enriched in OSCC lesions, while *Proteobacteria*, *Firmicutes* and *Actinobacteria* were considerably diminished (**Figure 4B****)**. At the genus level, 22 taxa including *Serratia*, *Anoxybacilus*, *Stenotrophomonas*, *Sutterella*, *Actinomyces*, *Bacillus*, *Lysobacter*, *Paenibacillus*, *Ammoniphilus*, *Bifidobacterium*, *Megamonas*, *Collinsella*, *Brevibacillus*, *Megasphaera*, *Blautia*, *Methylobacterium*, *Prevotella_9*, *Roseburia*, *Phenylobacterium*, *Pseudopropionibacterium*, *Parabacteroides*, and *Anaerobacillus* were significantly declined in the OSCC lesions as compared to the healthy controls (**Figure 4C****)**. In contrast, only five taxa including *Prevotella_7*, *Corynebacterium1*, *Pseudomonus*, *Deinococcus*, and *Noviherbaspirillum* were significantly enriched in the OSCC lesions as compared to the anatomically matched control samples (**Figure 4C****)**. Box-Whisker dot plots along with the pair-wise genus enrichment analysis were also performed to clearly visualize the differential enrichment pattern of top five bacterial genera identified by the LEfSe analyses between OSCC lesions and contralateral anatomically matched healthy controls (**Figure S3****)**.

In addition, a ‘Random Forest’ algorithm was employed to further assess the diversity in bacterial community at the species level among the OSCC samples and anatomically matched healthy controls (**Figure S4A**). The decision trees extracted from the random forest classification identified distinct bacterial species in OSCC lesions when compared with the normal samples (**Figure S4A**). The results demonstrated that in the total of 50 OSCC samples, 34 samples exhibited unique and 16 samples showed overlapping species, while in normal counterparts, 30 samples exhibited unique and 20 samples showed overlapping species (**Figure S4A**). LEfSe analyses further revealed *Capnocytophaga*, unidentified *Micrococcaceae* and uncultured *Cornebacterium* 1 species were considerably enriched in OSCC lesions, 29 different species, however, mostly unidentified and uncultured species, were significantly declined as compared to the paired contralateral anatomically matched controls (**Figure S4B****)**. Recent studies suggested that 16S rRNA based sequencing technologies targeting one or more hypervariable regions allow reliable identification of bacterial genera, but can potentially misguide identification of bacterial species (49, 50). In agreement to this, our study also demonstrated that the sequencing depth was not sufficient to accurately identify the oral microbial composition at the species level responsible for OSCC development. Therefore, to nullify the false positives at the species level, we have limited our analyses up to genus level for further investigation and subsequent conclusion.

### Functional Prediction of Oral Microbiome Associated With OSCC Development

**Figure 5:**
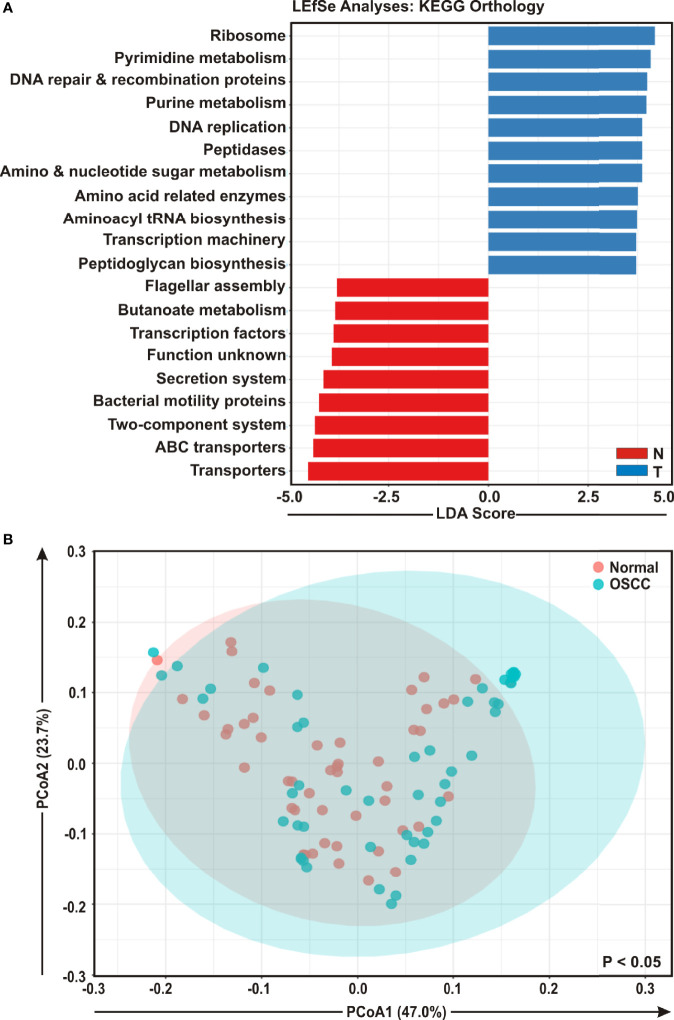
Functional analyses of identified microbial compositions in cancerous lesions and clinically normal samples. **(A)** Linear Discrimination Analysis (LDA) scores predicting gene function enriched among two different groups of oral squamous cell carcinoma (OSCC) lesions and normal samples using Phylogenetic Investigation of Communities by Reconstruction of Unobserved States (PICRUSt) according to the Kyoto Encyclopedia of Genes and Genomes (KEGG) database. **(B)** Principal Component Analysis (PCoA) of bacterial functions associated OSCC lesions and contralateral matched controls. Axis 1 (PCoA1): 47.0% of variation explained. Axis 2 (PCoA2): 23.7% of variation explained.

To envisage oral microbial functions connected to the development of OSCC, we employed the Phylogenetic Investigation of Communities by Reconstruction of Unobserved States (PICRUSt) (45) and accordingly Kyoto Encyclopedia of Genes and Genomes (KEGG) pathways (46) were generated specific for OSCC lesions and anatomically matched healthy controls (**Figure 5****)**. The LEfSe outputs demonstrated that function related to nucleotide synthesis and maintaining the fundamental functions of a cell such as pyrimidine and purine metabolism, DNA repair and recombination proteins, DNA replication, transcription machinery, amino and nucleotide sugar metabolism, protein translation related function such as ribosome, amino acid related enzymes, aminoacyl tRNA biosynthesis, peptidases, as well as peptidoglycan biosynthesis were associated with the progression of OSCC (**Figure 5A****)**. In contrast, parameters related to flagellar assembly, butanoate metabolism, secretion system, bacterial motility proteins, two-component system and ABC transporters, were inversely associated with OSCC development (**Figure 5A****)**. PCoA analyses also demonstrated that the predicted functions of bacterial compositions in two groups – OSCC lesions and the anatomically matched controls were significantly clustered (*p* < 0.05) (**Figure 5B****)**.

### Quantitative PCR Profile of Oncogenic Viruses Revealed Significant Association of HPV16 With OSCC Development

**Figure 6:**
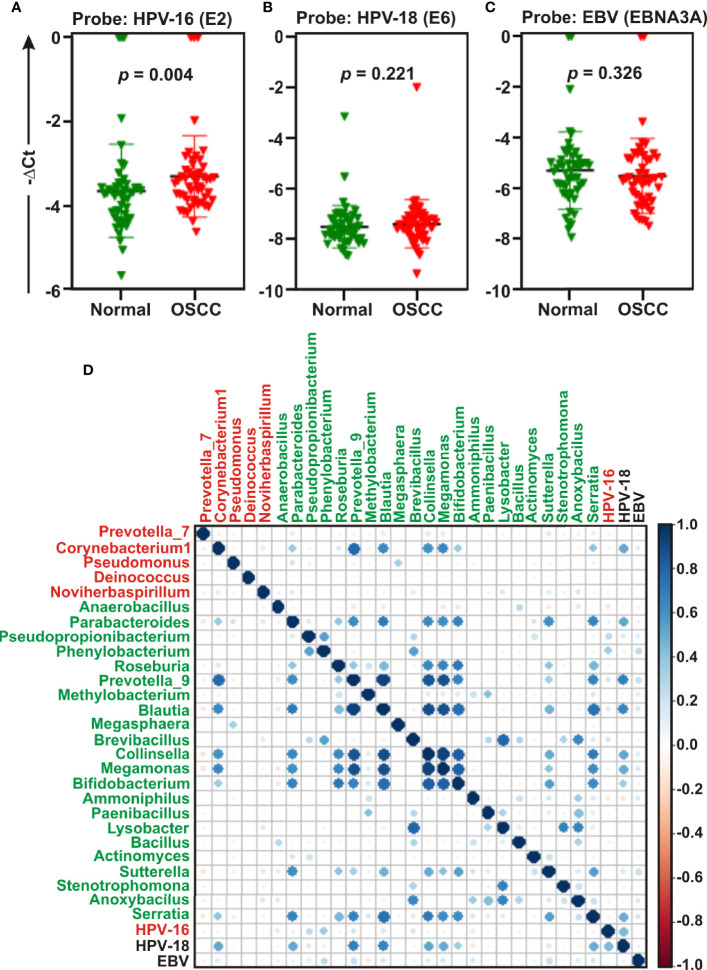
Quantitative PCR (qPCR) and co-occurrence analysis of human oncogenic viruses with identified bacterial genera in normal and oral squamous cell carcinoma (OSCC) samples. Comparative qPCR data of **(A, B)** high risk human papilloma viruses (HPVs) - **(A)** HPV-16 **(B)** HPV-18 and **(C)** Epstein-Barr virus (EBV). PCR calculation was performed by −ΔCT method to quantify relative abundance of each tumor virus using human genomic GAPDH as control. The -ΔC_t_ values of each sample were plotted using GraphPad Prism 8.0.1. **(D)** Pearson correlations among human oncogenic viruses and the top 27 most abundant bacterial genera identified by Linear Discrimination Analysis (LDA) Effect Size (LEfSe) analyses were calculated and analyzed. Correlation values range from −1.00 (red) to 1.00 (blue).

Studies suggest that a number of human oncogenic viruses including human papilloma viruses (HPVs) and Epstein-Barr virus (EBV) are associated with OSCC development (13–15). To assess the potential involvement of viral etiology in our samples we designed real time PCR primers against EBV encoded EBNA3A oncogene (GeneID: 3783762) along with two high risk HPV isotypes HPV-16 encoded E2 oncogene (GeneID: 1489080) and HPV-18 encoded E6 oncogene (GeneID: 1489088) and subsequently employed in quantitative PCR (qPCR) analyses (**Figures 6A–C****)**. The housekeeping gene human GAPDH gene was utilized as control assuming the genomic segment bearing GAPDH gene remained unaffected in both normal and OSCC affected tissue sections. A higher negative -ΔC_t_ ****(average GAPDH C_t_ value – average target primer C_t_ value) indicated elevated presence of the virus in the sample as detected by specific primer set targeting specific viral gene. Our results clearly demonstrated that only HPV-16 (*p* = 0.004) was significantly associated with OSCC lesions as compared to the control tissue sections (**Figure 6A****)**. In contrast, no significant association for both HPV-18 (*p* = 0.221) and EBV (*p* = 0.326) between the two sample groups was observed (**Figures 6B, C**, respectively). However, -ΔC_t_ ****values for both HPV-18 and EBV were higher than that of HPV-16, indicating a higher prevalence in oral tissue samples (compare **Figures 5B, C** with **5A**). Altogether these oncogenic viruses might regulate the onset as well as progression of oral cavity oncogenesis and thereby demands their detection along with bacterial dysbiosis.

Next, the co-occurrence and co-exclusion patterns of these oncogenic viruses with the 27 most abundant bacterial genera identified in LEfSe analyses in each group of OSCC lesions and contralateral matched controls were further investigated (**Figure 6D**). Overall, there was no negative correlation found in our analyses. In matched normal controls, *Prevotella_9* was found to be positively correlated with a number of bacterial genera. For example, *Prevotella_9* and *Blautia* were the most positively correlated (ρ = 0.926), followed by *Megamonas* (ρ = 0.872), *Collinsella* (ρ = 0.852), *Serratia* (ρ = 0.679), *Bifidobacterium* (ρ = 0.673), and *Parabacteroides* (ρ = 0.626). HPV-18 demonstrated moderate positive correlation with most of these genera – including *Prevotella_9* (ρ = 0.683), *Blautia* (ρ = 0.660), *Megamonas* (ρ = 0.509), *Collinsella* (ρ = 0.527), and *Parabacteroides* (ρ = 0.514) (**Figure 6D**, **Table S3****)**. In contrast, HPV-16 demonstrated no positive correlation with any bacterial genera identified in cancerous lesions (**Figure 6D****)**. Although *Corynebacterium1* was significantly associated with OSCC lesions, it demonstrated positive correlation with several bacterial taxa abundantly enriched in normal samples (**Figure 6D****)**. Among these, the most positively correlated genera were *Corynebacterium1* and *Prevotella_9* (ρ = 0.785) followed by *Megamonas* (ρ = 0.657), *Blautia* (ρ = 0.643), and *Collinsella* (ρ = 0.601) (**Figure 6D**, **Table S3**).

## Discussion

In spite of the highest oral cancer incidence, accounting to 30% of all cancers in India (6, 7), so far there are no reports describing changes of oral microbiome in OSCC among Indian patients. The purpose of the current investigation was to profile the dysbiosis of oral microbiota between OSCC lesions and contralateral anatomically matched control tissue samples prospectively collected from fifty patients of eastern region of India. In agreement with Guerrero-Preston et al. study (51), we also observed a significant loss in richness and diversity of oral bacterial communities in OSCC lesions compared to matched controls. However, several reports revealed enhanced diversity of bacterial communities in OSCC samples (31, 52, 53). Nevertheless, dysbiosis of oral microbiome appears to be strongly associated with OSCC development. Overall, the results demonstrated that *Prevotella, Corynebacterium*, *Pseudomonas*, *Deinococcus*, and *Noviherbaspirillum* genera were significantly enriched, while genera including *Actinomyces*, *Sutterella*, *Stenotrophomonas*, *Anoxybacillus*, and *Serratia* were depleted in the OSCC lesions as compared to the matched healthy controls.

Previously, several models of microbial infection and potential oral microbiome signature link to the pathology of a number of oral diseases including cancer have been established. For example, certain oral bacterial pathogens, including *Porphyromonas gingivalis* and *Fusobacterium nucleatum* have been reported to disrupt the equilibrium of oral microbiome and along with deregulated immune response eventually initiate periodontal diseases (periodontitis) (54–56). These well studied periodontal organisms subsequently prompted researchers to further investigate the precise role of dysbiotic oral micrbiota in developing oral cancer (57, 58). In general, our results agreed with the previously published data of enriched and depleted microbes associated with the OSCC development. Overall, five of the most abundant phyla including *Proteobacteria*, *Firmicutes*, *Actinobacteria*, *Bacteroidetes*, and *Fusobacteria* identified in our study were consistent with those found in previous studies. However, the less abundant phyla including *Tenericutes*, *Deinococcus*, and *Patescibacteria* detected were significantly varied among multiple studies. In addition, in line with the previous studies (29, 52, 53) *Firmicutes* was also found as the most abundant phylum in overall oral microbiome in our study. Of the significantly elevated genera in cancerous lesions, *Prevotella* and *Pseudomonus* were previously shown to be highly abundant in both periodontitis and OSCC samples when compared to healthy controls (32, 53, 59). Importantly, periodontitis has been suggested as a self-governing risk factor for OSCC development (27). Interestingly, in contrast to our finding, *Corynebacterium* was previously found to be decreased in oral cavity cancer (OCC) and oropharyngeal cancers (OPC) (60). Our results indicated presence of a unique genus *Deinococcus* although relatively less abundantly only in cancerous lesions and could not be detected in control tissue sections. Since in our study design, paired OSCC lesion and control tissue samples were obtained from single patient, it nullified the possibilities of inter-individual variation. Thus even small differences of bacterial communities among two these groups would represent significance in OSCC development. Species of the *Deinococcus* genus are recognized for their extreme resistance to ionizing radiation and oxidative stress and other damaging conditions (61). Although a number of earlier studies indicated the presence of *Deinococcus* genus (62, 63), the precise role of the members of this genus in OSCC is yet to be defined.

In our study, although *Fusobacteria* was identified as one of the most abundant phyla in overall oral bacteriome, its abundance showed no significant difference between OSCC lesions and normal tissue samples. This is in contrast to a number of recent reports which demonstrated significant abundance of several members of *Fusobacteriem* in OSCC lesions when compared to normal samples (31, 32). Mager et al. detected *F. periodonticum* in the saliva sample from OSCC patients using specific bacteria probes, but its abundance showed no significantly difference between OSCC-positive and OSCC-free patients (30). Yang et al. determined significant elevation of *F. periodonticum* species in OSCC lesions, whereas no significant difference was observed in case of *F. nucleatum* between tumor and normal samples (52). In contrast, Al-Hebshi *et al*. indicated that *F. nucleatum* was the most significantly enriched species in OSCC lesions as compared to the control normal tissues (32). The diverse presence of different members of *Fusobacterium* species identified in OSCC samples in multiple studies possibly arose due to varied sample types as well as subjects recruited of different ethnicity across the world. In addition, *Fusobacterium nucleatum* was also identified as one of the highly enriched bacterial species in colorectal cancer (64). Moreover, Komiya et al. showed that patients with colorectal cancer (CRC) have identical strains of *Fusobacterium nucleatum* in their CRC tissue section and oral cavity (65). Given the importance of *Fusobacterium* in various human cancers, further in depth investigation is required to verify *Fusobacterium* association with OSCC in Indian scenario with larger patients sample size.

Nucleotide metabolism is an important pathway that provides purine and pyrimidine molecules for DNA replication, RNA biogenesis, as well as cell bioenergetics. Increased nucleotide metabolism supports uncontrolled proliferation of cancer cells and thus represents a hallmark of cancer (66). Apart from nucleotide metabolisms, several critical pathways like DNA repair, recombination, protein synthesis and transcription machineries are frequently altered in tumor cells (67–69). Moreover, inhibitors that specifically blocks DNA replication and induce DNA damages have been widely used as chemotherapeutic agents against numerous cancers (70). In agreement to this, our PICRUSt analyses showed that function related to nucleotide metabolisms including both purine and pyrimidine synthesis as well as basic cell functions like DNA repair and replication and functions related to mRNA translation including ribosome, amino acid related enzymes, aminoacyl tRNA biosynthesis and peptidases were significantly linked to OSCC development. Although Yost et al. using metatranscriptomic analyses suggested importance of these pathways for OSCC development (71), so far there are no robust studies that directly linked microbes with these pathways in a tumor microenvironment. Moreover, in contrast to our study, Yang et al. demonstrated that parameters related to protein and amino acids metabolisms were inversely associated with OSCC progression from stage 1 to stage 4 patients (52). Previously a number of reports demonstrated that pathways related to bacterial chemotaxis and flagellar assembly were remarkably enriched in the OSCC group (32, 59). However, in contrast, our study showed that pathways related to flagellar assembly and bacterial motility proteins were inversely associated with the OSCC development.

A growing body of evidence suggested a potential association of several human tumor viruses with oral cancers (13–15, 72). For example, while low risk HPV subtypes including HPV-6 and HPV-11 are associated with a variety of oral benign papillomatous lesions such as oral squamous papilloma, oral verruca vulgaris, oral condyloma accuminatum and focal epithelial hyperplasia, high risk HPV subtypes including HPV-16 and HPV-18 are associated with malignant lesions (72–75). The transformation of normal oral mucosa in OSCC is potentially linked to precancerous lesions, such as OLP (76, 77). Although the precise role of viral mediated malignant transformation of precancerous lesions is not clear, HPV infection is significantly associated with OLP (72, 78). Overall, previous studies suggested that HPV-16 is the most frequently detected HPV subtype in oral cancers (79) and accordingly in 2012 the International Agency of Research of Cancer (IARC) acknowledged the significant association of HPV-16 high risk group with oral cancer development (14). In agreement to this, our results also demonstrated that HPV-16 but not HPV-18 was significantly associated with OSCC lesions as compared to anatomically matched control tissue sections.

In sum, using a carefully controlled patients’ cohort, herein we identified specific microbial signature associated with OSSCC development. However, the current study has several limitations including constraints associated with the 16S rRNA gene amplification based sequencing technologies (49, 50). Recent studies suggested although more than 99% of sequencing reads could be correctly classified at the genus level, a significant proportion at the species level might be misclassified during identification of bacterial populations by targeting various variable regions of the 16S rRNA gene. In agreement to this, our study also showed that the sequencing depth was not adequate to precisely classify the oral microbiota at the species level and thus, in order to nullify false positives, we restricted our analyses up to genus level. Another limitation of this study was relatively smaller sample size. In future, to validate the results a larger sample size with distinct cancer stages among population in different regions and different socioeconomic background would be highly preferable. We additionally lacked information on the involvement of other organisms particularly fungus and the association of different viruses with OSCC development. Strategies such as whole genome sequencing and metabolomics would allow in identification of changes of overall oral microbiota and their metabolities during OSCC development. Moreover, whole genome shotgun sequencing (metagenomics) would further validate the functional inferences from 16S rRNA amplicon sequences obtained using PICRUSt. Since the current study was conducted using tissue biopsy samples, it would be interesting to investigate whether the results could be extended in a non-invasive method by utilizing saliva samples. Altogether, longitudinal research activities are greatly demanded to explore the functional implications of the oral microbiota in terms of diagnosis and risk assessment of OSCC development, as well as potential expansion of current therapeutic strategies to restore the health of the oral ecosystem.

## Data Availability Statement

The 16S rRNA amplicon sequencing data from this study have been deposited in the NCBI BioProject under accession number PRJNA666746.

## Ethics Statement

The studies involving human participants were reviewed and approved by the Institutional Ethics Committee for Human Research, Indian Statistical Institute, Kolkata, India. The patients/participants provided their written informed consent to participate in this study.

## Author Contributions

PS and AS wrote the main manuscript text. PS, SM, SL, RC, and AS performed the bioinformatic analysis. PS and SB performed the experiments. JR recruited the patients and conducted the histopathological studies. SD and RC collected the samples and performed sampling. AS conceived, designed, and successfully sought funding for the study. All authors contributed to the article and approved the submitted version.

## Funding

This study was supported by grants from DBT/Wellcome Trust India Alliance Intermediate Fellowship research grant [IA/I/14/2/501537] and Science & Technology and Biotechnology, Govt. of West Bengal [1798 (Sanc.)/ST/P/S&T/9G-5/2019] to A.S. The funders had no role in study design, data collection and analysis, decision to publish, or preparation of the manuscript.

## Conflict of Interest

The authors declare that the research was conducted in the absence of any commercial or financial relationships that could be construed as a potential conflict of interest.

## Supplementary Material

Supplementary Figure 1 Preliminary quantitative PCR (qPCR) analyses of four OSCC and anatomically matched normal samples. PCR calculation was performed by −ΔCT method to quantify relative abundance of overall bacteria using primers against the conserved region of 16S rRNA gene and human genomic GAPDH gene segment as control. The -ΔC_t_ values of each sample were plotted using GraphPad Prism 8.0.1.

Supplementary Figure 2 Differentially abundant genera in OSCC lesions and anatomically matched normal samples. Relative abundance (%) of the taxa at the genus level in **(A)** all samples, **(B)** anatomically matched control tissues and **(C)** OSCC lesions. **(D)** Venn diagram depicts distinct and overlapped genera among OSCC and normal samples.

Supplementary Figure 3 Top five taxa at genus level identified in the LEfSe analysis among OSCC lesions and healthy matched controls. Box Whisker Plot (top) and Pair-wise (bottom) genus enrichment analysis of top five bacterial genera identified in LEFSe analysis as described in **Figure 4**among **(A)** normal samples and **(B)** OSCC lesions.

Supplementary Figure 4 Bacterial composition at the species level between paired OSCC lesions and contralateral normal tissue samples. **(A)** The error plots identified from random forest classification analyses. Red-line indicates the overall species present in both OSCC and normal samples, green-line indicates the distinct species present in the normal samples and the blue-line indicates the specific species present in the OSCC lesions. **(B)** Linear discriminant analysis effect size (LEfSe) analysis demonstrating differential bacterial composition at the species level between the OSCC lesions and anatomically matched healthy controls.

Supplementary Table 1 Clinical characteristics of patients’ samples.

Supplementary Table 2 Real time PCR primers.

Supplementary Table 3 Pearson correlations among HPV-16, HPV-18, EBV and the top 27 most abundant bacterial genera identified by LEfSe analyses.
