# Supplementary figures and images for: Dysbiosis of Oral Microbiota During Oral Squamous Cell Carcinoma Development
Source: Front Oncol. 2021 Feb 23;11:614448. doi: 10.3389/fonc.2021.614448 (PMC7940518; doi:10.3389/fonc.2021.614448)

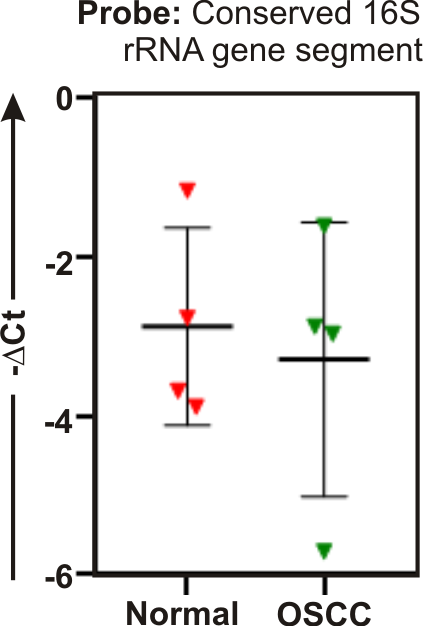

Supplement: Supplementary Figure 1 — Preliminary quantitative PCR (qPCR) analyses of four OSCC and anatomically matched normal samples. PCR calculation was performed by −ΔCT method to quantify relative abundance of overall bacteria using primers against the conserved region of 16S rRNA gene and human genomic GAPDH gene segment as control. The -ΔCt values of each sample were plotted using GraphPad Prism 8.0.1. [file Image_1.tif]

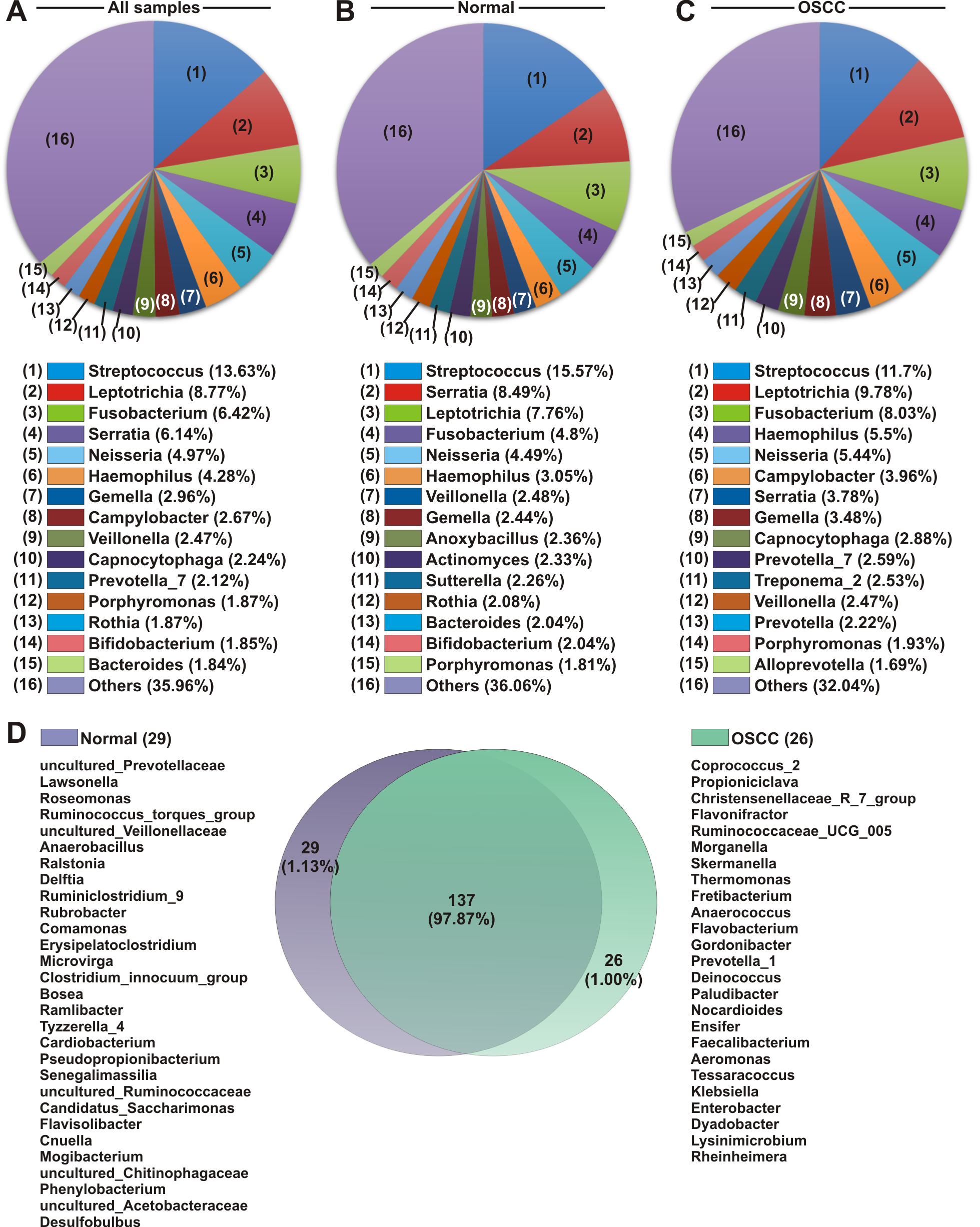

Supplement: Supplementary Figure 2 — Differentially abundant genera in OSCC lesions and anatomically matched normal samples. Relative abundance (%) of the taxa at the genus level in (A) all samples, (B) anatomically matched control tissues and (C) OSCC lesions. (D) Venn diagram depicts distinct and overlapped genera among OSCC and normal samples. [file Image_2.tif]

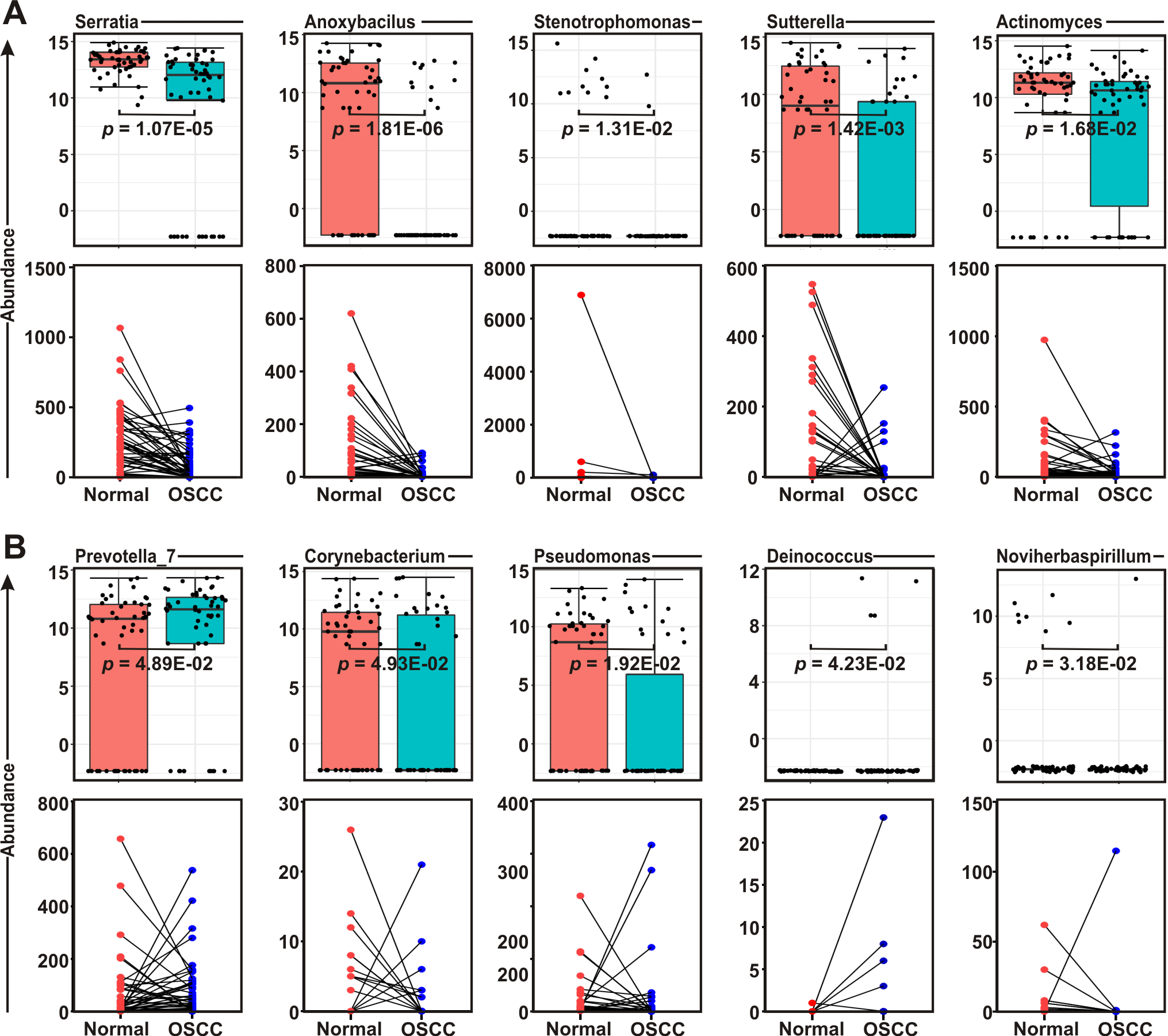

Supplement: Supplementary Figure 3 — Top five taxa at genus level identified in the LEfSe analysis among OSCC lesions and healthy matched controls. Box Whisker Plot (top) and Pair-wise (bottom) genus enrichment analysis of top five bacterial genera identified in LEFSe analysis as described in Figure 4among (A) normal samples and (B) OSCC lesions. [file Image_3.tif]

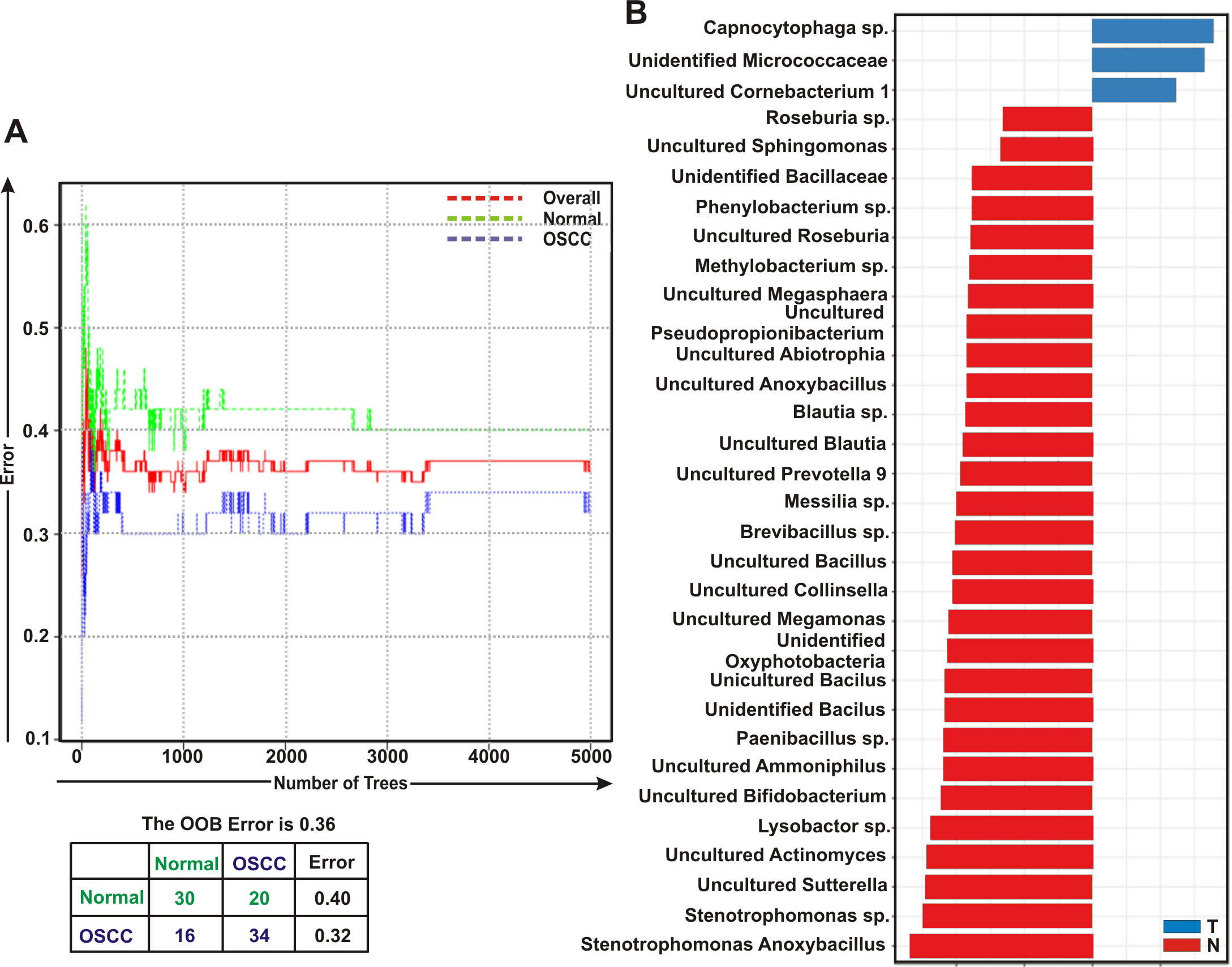

Supplement: Supplementary Figure 4 — Bacterial composition at the species level between paired OSCC lesions and contralateral normal tissue samples. (A) The error plots identified from random forest classification analyses. Red-line indicates the overall species present in both OSCC and normal samples, green-line indicates the distinct species present in the normal samples and the blue-line indicates the specific species present in the OSCC lesions. (B) Linear discriminant analysis effect size (LEfSe) analysis demonstrating differential bacterial composition at the species level between the OSCC lesions and anatomically matched healthy controls. [file Image_4.tif]
