# Supplementary material for: Dysbiosis of Oral Microbiota During Oral Squamous Cell Carcinoma Development
Source: Front Oncol. 2021 Feb 23;11:614448. doi: 10.3389/fonc.2021.614448 (PMC7940518; doi:10.3389/fonc.2021.614448)
Supplement: Supplementary Table 1 — Clinical characteristics of patients’ samples. [file Table_1.docx]

**Table S1. Clinical characteristics of patients’ samples.**

| **Sl**  **No.** | **Sample ID** | **Age** | **Sex** | **Clinical Diagnosis** | **Histopathological Diagnosis** | **Oral Habits** |
| --- | --- | --- | --- | --- | --- | --- |
|  |  |  |  |  |  |  |
| 1 | S1 | 37 | M | Oral Carcinoma | Well differentiated Squamous Cell Carcinoma | Pan masala (20-30/day) for 7-8 years |
| 2 | S2 | 60 | F | Malignancy with Verrucous Carcinoma | Well differentiated Squamous Cell Carcinoma | Pan masala chewing 4-5 times/day , areca nut chewing daily 4-5 packets for last 35 years. |
| 3 | S3 | 54 | F | Malignancy | Well differentiated Squamous Cell Carcinoma | No Habits |
| 4 | S4 | 36 | M | Malignancy | Well differentiated Squamous Cell Carcinoma | Gutkha (10-15 sachet/day for 2 years) |
| 5 | S5 | 62 | F | Malignancy | Well differentiated Squamous Cell Carcinoma | Pan masala chewing (10-12packets/day)since last 30 years |
| 6 | S6 | 70 | F | Verrucous Carcinoma | Well differentiated Squamous Cell Carcinoma | Pan, khaini, tobbaco chewing for more than 20 years |
| 7 | S7 | 48 | M | Tongue Carcinoma | Well differentiated Squamous Cell Carcinoma | No Habits |
| 8 | S8 | 44 | M | Malignancy(recurrence case of maligancy) | Well differentiated Squamous Cell Carcinoma | Pan masala and jarda, but patient had left habit 2 years back when he was diagnosed with malignancy |
| 9 | S9 | 80 | F | Malignancy | Well differentiated Squamous Cell Carcinoma | Snuff rubbing daily for than 25 years |
| 10 | S10 | 42 | F | Malignancy | Well differentiated Squamous Cell Carcinoma | Gurakhu twice a day For more than 20 years |
| 11 | S11 | 63 | M | Malignancy | Well differentiated Squamous Cell Carcinoma | No Habits |
| 12 | S12 | 45 | M | Malignancy | Well differentiated Squamous Cell Carcinoma | No Habits |
| 13 | S13 | 35 | M | OSMF with Malignancy | Well differentiated Squamous Cell Carcinoma | Khaini 8-10 times a day for more than 20 years, areca nut chewing and cigarette smoking occasionally |
| 14 | S14 | 50 | M | Malignancy | Well differentiated Squamous Cell Carcinoma | Pan chewing |
| 15 | S15 | 60 | M | Malignancy | Well differentiated Squamous Cell Carcinoma | Pan chewing 15 times a day |
| 16 | S16 | 62 | M | Malignancy | Well differentiated Squamous Cell Carcinoma | Bidi smoking 1 packet/day for more than 35 days |
| 17 | S17 | 55 | M | Malignancy | Well differentiated Squamous Cell Carcinoma | Khaini chewing for more than 30 years |
| 18 | S18 | 50 | F | Maligncy | Well differentiated Squamous Cell Carcinoma | Placing 'Gurakhu' at the site of the lsion 3-4 times / day for 15 mins / placement |
| 19 | S19 | 45 | F | Malignancy | Well differentiated Squamous Cell Carcinoma | Pan chewing (15/day) |
| 20 | S20 | 55 | F | Malignancy | Well differentiated Squamous Cell Carcinoma | No Habits |
| 21 | S21 | 40 | M | Tongue Carcinoma | Well differentiated Squamous Cell Carcinoma | Pan masala & jarda Supari chewing |
| 22 | S22 | 65 | F | Malignancy | Well differentiated Squamous Cell Carcinoma | Tobacco chewing for 30 years, quitted last 10 years |
| 23 | S23 | 73 | M | Malignancy | Well differentiated Squamous Cell Carcinoma | Gudaku brushing |
| 24 | S24 | 60 | M | Malignancy | Well differentiated Squamous Cell Carcinoma | Bidi, Quitted last 5-6 years |
| 25 | S25 | 60 | M | Malignancy | Well differentiated Squamous Cell Carcinoma | No Habits |
| 26 | S26 | 60 | M | Malignancy | Well differentiated Squamous Cell Carcinoma | No Habits |
| 27 | S27 | 32 | M | Malignancy | Well differentiated Squamous Cell Carcinoma | No Habits |
| 28 | S28 | 45 | M | Malignancy | Well differentiated Squamous Cell Carcinoma | No Habits |
| 29 | S29 | 60 | M | Malignancy | Well differentiated Squamous Cell Carcinoma | No Habits |
| 30 | S30 | 40 | F | Malignancy | Well differentiated Squamous Cell Carcinoma | Pan masala chewing with supari occassionally since 20 years |
| 31 | S31 | 61 | M | Malignancy | Well differentiated Squamous Cell Carcinoma | Pan and Khaini |
| 32 | S32 | 45 | F | Malignancy | Well differentiated Squamous Cell Carcinoma | No Habits |
| 33 | S33 | 47 | M | Malignancy | Well differentiated Squamous Cell Carcinoma | Gutka (10-15 packets/day since 15 years) and alcohol 120 ml / day since 6-7 years |
| 34 | S34 | 30 | F | Malignancy | Well differentiated Squamous Cell Carcinoma | Pan,tobacco(6/day) |
| 35 | S35 | 59 | M | Malignancy | Well differentiated Squamous Cell Carcinoma | Tobacco chewing since 10years |
| 36 | S36 | 44 | M | Erythroplakia | Well differentiated Squamous Cell Carcinoma | Pan (4-5/day) and bidi (4/day) for last 20 years |
| 37 | S37 | 38 | M | Malignancy | Well differentiated Squamous Cell Carcinoma | Khaini (20 times/day) and pan with jarda (6-8/day) for ~15 years |
| 38 | S38 | 60 | M | Malignancy | Well differentiated Squamous Cell Carcinoma | Pan masala 8-10 packets/day &bidi 1packet/day smoking since more than 25 years (left habitsince 2 months) |
| 39 | S39 | 62 | F | Malignancy | Well differentiated Squamous Cell Carcinoma | Pan chewing 2-3/day |
| 40 | S40 | 65 | F | Malignancy | Well differentiated Squamous Cell Carcinoma | Pan chewing 8-9/day for more than 25 years |
| 41 | S41 | 65 | F | Malignancy | Well differentiated Squamous Cell Carcinoma | Nassi intake |
| 42 | S42 | 41 | M | Verrucous Carcinoma | Well differentiated Squamous Cell Carcinoma | No Habits |
| 43 | S43 | 52 | F | Malignancy | Well differentiated Squamous Cell Carcinoma | Gurakhu rubbing regularly for more than 15 years |
| 44 | S44 | 56 | M | Malignancy | Well differentiated Squamous Cell Carcinoma | Pan masala chewing 7-8 packets daily |
| 45 | S45 | 42 | M | Malignancy | Well differentiated Squamous Cell Carcinoma | Bidi smoking (7-8/day), Khaini (3-4packets /day), both for more 20 years |
| 46 | S46 | 40 | M | Verrucous Hyperplasia | Well differentiated Squamous Cell Carcinoma | Khaini for more than 15 years |
| 47 | S47 | 55 | M | Malignancy | Well differentiated Squamous Cell Carcinoma | Bidi smoking 1 packet (25 pieces) per day for more than 20 years |
| 48 | S48 | 57 | M | Malignancy | Well differentiated Squamous Cell Carcinoma | Khaini And pan masala 4-6/day for more than 25 years |
| 49 | S49 | 65 | M | Malignancy | Well differentiated Squamous Cell Carcinoma | No Habits |
| 50 | S50 | 62 | F | Malignancy | Well differentiated Squamous Cell Carcinoma | Pan chewing along with tobacco |
