# Supplementary material for: Dysbiosis of Oral Microbiota During Oral Squamous Cell Carcinoma Development
Source: Front Oncol. 2021 Feb 23;11:614448. doi: 10.3389/fonc.2021.614448 (PMC7940518; doi:10.3389/fonc.2021.614448)
Supplement: Supplementary Table 2 — Real time PCR primers. [file Table_2.docx]

**Table S2. Real time PCR primers.**

| **Sl No.** | **Name of the Organism** | **Gene Segment** | **Forward Primer** | **Reverse Primer** |
| --- | --- | --- | --- | --- |
| 1 | Human papillomavirus 16 (HPV-16) | E2 | 5’-CAGACGACTATCCAGCGACC-3’ | 5’-GCAGTGAGGATTGGAGCACT-3’ |
| 2 | Human papillomavirus 18 (HPV-18) | E6 | 5’-AAGCTCAGCAGACGACCTTC-3’ | 5’-CCCTCCCCGTCTGTACCTTA-3’ |
| 3 | Epstein-Barr virus (EBV) | EBNA3A | 5’-GCCCTGGATGACAACATGGA-3’ | 5’-CAGGTGGGCATCTTCTGCTT-3’ |
| 4 | Bacteria | 16S rRNA V3  conserved domain | 5’-CCTACGGGAGGCAGCAG-3’ | 5’-ATTACCGCGGCTGCTGG-3’ |
| 5 | Human Glyceraldehyde 3-phosphate dehydrogenase (GAPDH) gene | GAPDH | 5’-AGCTGGCCCGATTTCTCCTC-3’ | 5’-ATGACTCAGCTTCTCCCGGC-3’ |
